# A hereditary disposition for bovine peripheral nerve sheath tumors in Danish Holstein cattle

**DOI:** 10.1186/s13028-014-0085-8

**Published:** 2014-12-10

**Authors:** Anette B Grossi, Jørgen S Agerholm, Knud Christensen, Henrik E Jensen, Páll S Leifsson, Christian Bendixen, Peter Karlskov-Mortensen, Merete Fredholm

**Affiliations:** Department of Veterinary Disease Biology, Faculty of Health and Medical Sciences, University of Copenhagen, Ridebanevej 3, 1870 Frederiksberg C, Denmark; Department of Large Animal Sciences, Faculty of Health and Medical Sciences, University of Copenhagen, Dyrlægevej 68, 1870 Frederiksberg C, Denmark; Department of Veterinary Clinical and Animal Sciences, Faculty of Health and Medical Sciences, University of Copenhagen, Grønnegårdsvej 3, 1870 Frederiksberg C, Denmark; Department of Molecular Biology and Genetics, Faculty of Science and Technology, University of Aarhus, Blichers Allé 20, 8830 Tjele, Denmark; Present address: Ellegaard Göttingen Minipigs A/S, Sorø Landevej 302, 4261 Dalmose, Denmark

**Keywords:** Cattle, Genetics, Genome wide association study, Neoplasms, Neurofibroma, Neurofibromatosis, Schwannoma

## Abstract

**Background:**

Peripheral nerve sheath tumors (PNSTs) are frequently found in Danish cattle at slaughter. Bovine PNSTs share several gross and histopathological characteristics with the PNSTs in humans with heritable neurofibromatosis syndromes. The aim of the present study was to investigate a possible hereditary disposition to PNSTs in dairy cattle by statistical analysis performed on data from 567 cattle with PNSTs. Furthermore, a preliminary genome-wide association study (GWAS) was performed on DNA isolated from 28 affected and 28 non-affected Holstein cows to identify loci in the bovine genome involved in the development of PNSTs.

**Results:**

PNSTs were significantly more common in the Danish Holstein breed than in other breeds with 0.49% of Danish Holsteins slaughtered during an eight-year-period having PNSTs. PNSTs also occurred significantly more frequently in the offspring of some specific Holstein sires. Examination of three generation pedigrees showed that these sires were genetically related through a widely used US Holstein sire. The PNSTs included in GWAS were histologically classified as neurofibroma-schwannoma (43%), schwannoma (36%) and neurofibroma (21%) and derived from Holstein cows with multiple PNSTs. A single SNP on chromosome 27 reached genome-wide significance.

**Conclusions:**

Gross and histological characteristics of bovine PNSTs are comparable to PNSTs in humans (schwannomatosis). Danish Holsteins are genetically disposed to develop PNSTs but the examined materials are insufficient to allow determination of the mode of inheritance.

## Background

Several abattoir surveys of neoplasms in cattle have shown that bovine peripheral nerve sheath tumors (PNSTs) are among the three most common neoplasms in cattle [1-5]. Bovine PNSTs rarely give rise to clinical signs and are most commonly found in the brachial nerve plexus, heart, and intercostal and mediastinal nerves of old cows at the time of slaughter [3,4,6,7]. Depending on their histological and immunohistochemical characteristics, benign bovine PNSTs can be classified into schwannoma, neurofibroma, and hybrid neurofibroma-schwannoma [7]. The histomorphological and immunohistochemical characteristics of the subtypes of bovine PNSTs are comparable to human PNSTs with neurofibromatosis syndromes 1 and 2, and schwannomatosis [7]. In humans neurofibromatosis 1 and 2 (NF1 and NF2) are autosomal dominant genetic disorders, and half of all cases are inherited from a parent with NF1 or NF2 [8,9]. In contrast, more than 75% of the schwannomatosis cases occur sporadically [10]. NF1 is characterized by the onset of multiple clinical signs including the development of multiple cutaneous neurofibromas [11]. One of the key steps in the formation of neurofibromas is loss of NF1 tumor suppressor gene function in Schwann cells. The NF1 gene product neurofibromin facilitates the inactivation of Ras proteins that regulate cellular responses such as mitogenesis and migration [11]. NF2 is less common than NF1 and is characterized by schwannomas of the cranial and spinal nerve roots. Patients with NF2 typically present with uni- or bilateral vestibular schwannoma in addition to other tumor types like meningioma and glioma [8]. The NF2 gene, which is inactivated in patients with NF2, encodes a protein called Merlin of unknown function [8]. Finally, multiple schwannomas, hybrid neurofibroma-schwannomas and sometimes neurofibromas in peripheral and cranial nerves are characteristic of schwannomatosis [12,13]. In 40-50% of the familial and in 10% of the sporadic cases of schwannomatosis the tumor suppressor gene *SMARCB1* is involved in the pathogenesis [10].

A disorder resembling NF1 has been described in four Holstein cows from the same herd [14]. The cows had multiple cutaneous neurofibromas and three of them were from the same sire lineage. The same allele of an informative polymorphism at the NF1 locus was detected in two of the cows and their sire [14]. The observation that several animals from the same herd develop PNSTs has led to the speculation that bovine PNSTs might be induced by a virus [15,16]. In addition, some authors have found virus-like particles in Schwann cells and fibroblasts by ultrastructural examination of bovine PNSTs [16-18]. However, attempts to confirm the viral origin of the particles by virus isolation, immunohistochemistry or animal inoculations have been unsuccessful [16-18].

The aim of the present study was to investigate a possible hereditary disposition to PNSTs in cattle. Therefore we performed statistical analyses on data from 567 slaughtered dairy cattle diagnosed with PNSTs at *post mortem* inspection. Moreover, a preliminary genome wide association study (GWAS) on 28 cows with confirmed PNSTs and 28 cows with no pathological evidence of PNSTs was performed to identify loci in the bovine genome involved in the pathogenesis of PNSTs.

## Methods

The tissue specimens for microscopical examination and genetic analysis derived from 28 slaughtered Holstein cows diagnosed with PNSTs at the *post mortem* inspection. At the abattoir, specimens of neoplastic tissue from each animal were fixed in 10% neutral buffered formalin. In addition, samples from the spleen and PNSTs were stored at – 20°C. Spleen samples of 28 unaffected Holstein cows aged ≥ 9 years were also frozen and used as controls in the genetic analyses. Carcasses with PNSTs underwent deboning to disclose all neoplasms, and the ear tag number of the animal and the location of the PNSTs were recorded.

### Histological and immunohistochemical examinations

The formalin fixed specimens were processed conventionally and embedded in paraffin. From each sample, 2–3 μm sections were cut and stained with hematoxylin and eosin (HE) for histological assessment. For immunohistochemistry, sections were mounted on adhesive-coated slides (Superfrost®Plus; Menzel-Glazer, Braunschweig, Germany), processed through xylene, and rehydrated in ethanol. Antigen retrieval was done by boiling in a microwave oven (700 W) twice for 5 min in Tris-EDTA buffer (1.21 g TRIS base [A 1379; Applichem, Darmstadt, Germany] and 0.372 g EDTA buffer [8418; Merck, Darmstadt, Germany] to 1 liter of distilled water), pH 9 (S100 and NF); or 0.01 M citrate buffer, pH 6 (CNPase). Endogenous peroxidase and unspecific protein binding were blocked with 0.6% (v/v) H_2_O_2_ in TBS (pH 7.6) for 15 min at room temperature and with Ultra V block (Lab Vision, Thermo Fisher Science, Fremont, CA, USA) for 5 min at room temperature, respectively. The slides were incubated for 24 h at 4°C with primary antibodies to CNPase (clone 11-5B, Sigma-Aldrich) diluted 1:800; S100 (polyclonal, Dako Cytomation) diluted 1:5000; and NF (polyclonal, AbD Serotec) diluted 1:6000. The detection system Ultra Vision ONE, HRP polymer was applied in accordance with the manufacturer’s instructions (Lab Vision Thermo Fisher Scientific). The chromogen was 3-amino-9-ethylcarbazoloe (AEC-red) (Lab Vision Thermo Fisher Scientific), and the sections were counterstained with Mayer’s hematoxylin. Slides were given two 5 min washes in TBS at pH 7.6 before addition of each reagent. Normal bovine peripheral nerve tissue was used as positive controls. Negative controls with the primary antibody replaced by 1% bovine serum albumin in TBS, 5% normal swine serum in TBS, or with a nonsense monoclonal (matching isotype) or polyclonal antibody of the same concentration as the primary antibody were run in parallel.

### Statistical analysis

In the period 2003–2010, the unique ear tag number of all cattle diagnosed with PNSTs at the Danish Crown slaughterhouse in Tønder, Denmark was registered. A total of 669 animals were registered as having PNSTs, and the following data were obtained from the Danish Cattle Database on all animals: age, sex, breed, herd location at birth, herd location immediately before slaughter, sire, paternal and maternal grandsires, date of latest calving, number of lactations, details of milk production (volume, fat and protein) during the preceding 305 days, and weight of carcass. Data on cattle with PNSTs were compared to data on all cattle slaughtered at the abattoir in 2003–2010 (n = 699,116). The statistical analyses were restricted to animals aged 5–12 years being of the Holstein, Red Danish Dairy and Jersey breeds (n = 144,432). However, all breeds were included in the statistical analysis of the effect of sex. When analyzing the effect of sire, the analyses were limited to Holsteins, due to the low occurrence of PNSTs in the other breeds, and the dataset was reduced to sires with more than five affected offspring aged 5–12 years. Furthermore, the age of the offspring was included in the model as a significant difference could be due to the longer life expectance of the offspring of some bulls in comparison with other bulls. Data were analyzed by the Chi-square test, Fisher’s exact test, or the General Linear Model procedure (SAS Institute, Cary, NC, USA). The GLM procedure was used applying the SAS model statements were *model PNST = age sire*, and *model PNST = age grand-sire* , where ‘age’, ‘sire’ and ‘grand-sire’ were defined as class variables. Additionally, a model including the production traits milk yield and weight at slaughter was applied.

### Genome-wide association study

Genomic DNA from 28 cases and 28 controls was isolated from the spleen using the salting out procedure [19] with modifications. Briefly, cells from 0.1-0.5 g tissue were lysed in 500 μL lysis buffer (100 mM Tris, 5 nM EDTA, 200 mM NaCl and 0.2% SDS) and 10 μL proteinase K for 24 h at 55°C, DNA was precipitated with 500 μL isopropanol and the pellet was washed with 70% ethanol and dissolved in 200 μL TE buffer (10 mM Tris, 1 mM EDTA), DNA was precipitated with 20 μL 5 M NaCL and 600 μL 100% ethanol and dissolved in 100 μL. Genotyping was performed on DNA samples diluted to 50 μg/μL at GenoScan A/S, Tjele, Denmark using the BovineSNP50 BeadChip. GWA was performed using PLINK [20] and all markers were subject to strict quality control; only SNPs with a minor allele frequency > 5%, a call rate of > 90% and in Hardy-Weinberg equilibrium in controls (*P* = 0.05) were included in subsequent analysis. All samples had less than 10% missing genotype calls. The threshold for genome-wide significance was set by a permutation test using 100,000 permutations. Multidimensional scaling (MDS) analysis was carried out using PLINK [20] and used to assess population stratification.

## Results

### Gross and histological examinations

**Figure 1:**
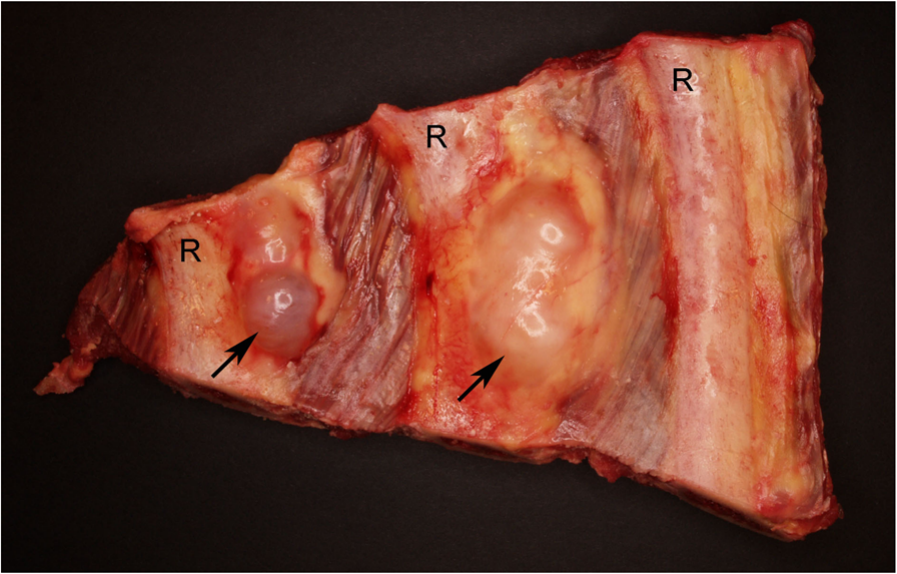
**Bovine peripheral nerve sheath tumors.** Two tumors (arrows) located in relation to intercostal nerves (not visible). Specimen displaying the internal surface of the thorax. R: rib.

The 28 Holstein cows included in the genetic analysis all presented with multiple PNSTs that were confined to the brachial nerve plexus (21), intercostal nerves (21) (Figure 1), mediastinal nerves (16), the heart (9), spinal nerve roots (2), and in lymph nodes (1).

The PNSTs were classified in accordance with the classification criteria proposed by Nielsen *et al.* [7] as hybrid neurofibroma-schwannoma (12/28), schwannoma (10/28) and neurofibroma (6/28). In schwannomas, the Antoni A pattern was predominant with closely packed spindle-shaped cells arranged in whorls, bundles or palisades with Verocay bodies. Smaller areas with Antoni B pattern characterized by rounded and loosely arranged cells were also seen. In schwannomas, strong, diffuse immunoreactivity for CNPase and S100 was characteristic. The neurofibromas were more heterogeneous, less cellular, and with cells organized more loosely into bundles or short interwoven fascicles. The immunolabeling for CNPase and S100 was focal and only seen in some of the neoplastic cells. NF positive axons were mainly seen in neurofibromas and areas of neurofibroma. Hybrid neurofibroma-schwannomas were composed of both schwannoma and neurofibroma. In the majority of the hybrid tumors the schwannoma component was predominating.

### Statistical analyses

PNSTs were diagnosed in 669 slaughter cattle (0.09%) during 2003–2010. Of these, 562 were Danish Holsteins and five were Red Danish Dairy cattle. The remaining cases were either beef cattle or crossbreds, while no cases were recorded in Jersey cattle. The statistical analyses were restricted to the Holstein and Red Danish Dairy breeds (n = 567) when relevant.

#### Breed

The animals surveyed included the three most common Danish dairy breeds: 115,248 Holsteins, of which 0.49% had PNSTs; 10,045 Red Danish Dairy, of which 0.05% had PNSTs; and 19,139 Jersey, of which none were affected. Thus, PNSTs were significantly more prevalent in the Holstein breed than in the other breeds (x^2^ = 223.62, df = 2, *P* < 0.0001).

#### Age

**Figure 2:**
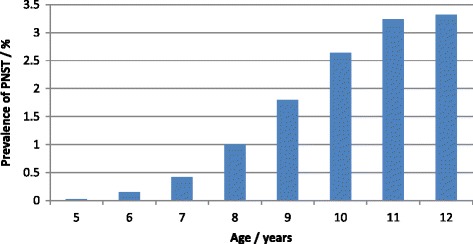
**Association between age and prevalence of peripheral nerve sheath tumors in Danish dairy cattle (n = 567) (Holsteins: n = 562; Danish Red: n = 5).**

The mean age at slaughter of cattle with PNSTs was 8.69 years (SD = 2.08) and was significantly higher in affected than non-affected cattle (χ^2^ = 1018.93, df = 7, *P* < 0.0001). Figure 2 shows the effect of age and prevalence of PNST in Holsteins and Red Danish Dairy cattle.

#### Sex

PNSTs were almost exclusively seen in females (99.7% of the cases), but this was probably caused by culling of males before PNSTs were macroscopically detectable or even before the usual age of tumor development. In cattle aged 5–12 years, there was no significant effect of sex (Fisher’s exact test, two-sided probability *P* = 0.7297).

#### Herd

A simple count showed that 1,590 herds had more than 4 animals with an age of more than five years of age. The affected Holstein cattle derived from 248 different herds of which only three had more than four PNST cases. Overall, 1,590 herds had delivered more than four animals aged at least four years.

#### Sire and maternal grandsire

A statistically significant higher number of affected offspring of some sires was observed even after correction for age (F value = 4.27, df = 13, *P* < 0.0001). By comparing the least squares means, four Holstein bulls appeared to have a higher prevalence of affected offspring than others. The prevalence of affected offspring for these four bulls was between 0.52% and 2.30% of the offspring slaughtered at the age of 5–12 years during the study period. Examination of three generation pedigrees showed that three of the four bulls were genetically related though a widely used US Holstein sire in generation three. In addition, a statistically significant effect of maternal grandsire was seen (F value = 2.78, df = 16, *P* = 0.0002) as five bulls had a relative higher prevalence of affected offspring than others (1.10%-2.67% of the offspring slaughtered at the age 5–12 years). Three of these also had the widely used US Holstein sire in their pedigree in generations 1–3.

#### Production

Presence of PNST at slaughter was not associated with weight at slaughter or milk yield.

### Genome-wide association study

Multidimensional scaling and QQ-plot did not indicate population stratification to be a special concern in the material (data not shown). The genomic inflation factor was 1.18, hence genomic correction (GC) was applied.

**Figure 3:**
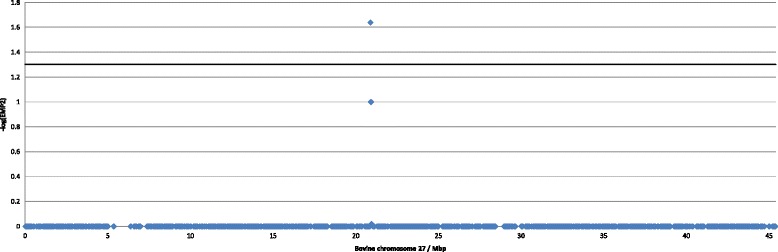
**Manhattan plot of genome-wide association study results.** X-axis: Position on bovine chromosome 27 in mega base pairs (Mbp) according to the UMD3 assembly. Y-axis: −log(EMP2), where EMP2 is the genome wide corrected empirical P-value determined based on 100,000 permutations. The horizontal line indicates the genome wide significance threshold. One SNP, HAPmap49086-BTA-22323 at 20.9 Mb, exceeds the genome wide significance level and the neighboring SNP approaches the genome wide significance level. All other SNPs on chromosome 27 and in the rest of the genome (data not shown) has a -log(EMP2) close to 0.

Genotyping success rate was > 98% and 40,962 SNPs passed quality check. All 56 individuals passed quality control. A full model association test with calculation of exact *P*-values was performed in PLINK using the options ‘--model’ and ‘--fisher’. Four SNPs were identified with point wise *P*-values (EMP1) of 0.000999 (Table 1). Only one SNP (HAPmap49086-BTA-22323) reached genome-wide significance after genomic correction (EMP1_GC_) and after correcting initial raw *P*-values for multiple hypotheses testing by permutation (EMP2). EMP1_GC_ = 7.88 × 10^−6^; EMP_Bonferroni_ = 0.049; EMP2 = 0.02298; Benjamini Hochberg False Discovery Rate (FDR_BH_) = 0.049. The neighboring SNP (ARS-BFGL-NGS-65900) also stands out compared to other SNPs on chromosome 27 but did not reach genome wide significance level (Figure 3). HAPmap49086-BTA-22323 is located at 20.9 Mb on chromosome 27 (UMD3). This SNP was identified under the allelic model and no other model resulted in significant associations.

**Table 1:** ***P*** **-values of SNPs reaching genome-wide significance**

**CHR**	**SNP**	**EMP1**	**EMP2**
27	Hapmap49086-BTA-22323	0.000999	*0.02298*
27	ARS-BFGL-NGS-65900	0.000999	0.0999

CHR: chromosome no; SNP: Single nucleotide polymorphism; EMP1: Initial empirical *P*-values; EMP2: Empirical *P*-values after correction for multiple hypothesis testing by permutation.

The genotype distribution of the chromosome 27 SNP reaching genome-wide significance was six AG and 22 GG animals among the controls and 10 AA, 10 AG, 8 GG among the PNST cases.

## Discussion

Several points of resemblance to patients with schwannomatosis were observed. As in humans [12], the animals presented with multiple schwannomas, hybrid neurofibroma-schwannomas and sometimes neurofibromas confined to spinal nerve roots and peripheral nerves. The majority of the human cases of schwannomatosis are sporadic and only in about 15-25% of the patients, the disease is inherited as an autosomal dominant trait [10]. In Danish Holstein cattle, the incidence of PNSTs was significantly higher than in the breeds Red Danish Dairy and Jersey, which are comparable regarding management practices like for example culling age. This indicates a hereditary disposition to PNSTs in Holsteins.

All cattle included in the study were slaughtered at one abattoir in the southern part of Denmark. We can therefore not extrapolate the prevalence to the entire Danish cattle population. A viral etiology of PNSTs in cattle has been proposed [17,18] but there are no data in our study that supports that hypothesis. Significant breed differences were observed, which points against an infectious etiology taking into consideration that the three major dairy breeds are held under comparable conditions and that affected cattle derived from 248 different farms and without variations over time.

In humans, sporadic schwannomas occur in individuals of all ages with a peak incidence between the third and sixth decades; whereas, the majority of individuals with familial schwannomatosis presents with clinical signs, usually chronic pain, at a younger age (second and third decade) [21]. In comparison, the incidence of PNSTs in cattle increased with age. Furthermore, there was no effect of PNSTs on weight at slaughter and milk yield, which would be expected in animals with chronic pain.

Analysis of pedigree data in the present study failed to show a clear Mendelian inheritance of PNSTs. However, a statistical significant effect of sires and maternal grandsires was found. Phenotypic information on PNST in the grandsires was not available. Some of the sires were rather closely related through a common ancestor, but this may simply be by chance, as some breeding lines are very frequently represented in the Holstein breed worldwide. As the incidence of PNSTs increases with age, it is possible that some of the animals included in the statistical analyses would have developed PNSTs if allowed to live longer. Thus, a Mendelian inheritance might have been identified if all the animals had been slaughtered at the age of 12 years.

The GWAS identified a single SNP at chromosome 27 associated with PNST. Additionally, this SNP and the neighboring SNPs were the only two SNPs in the study that stood out from the rest. The test for association to every other SNPs had a p-value close to 1. The result, however, still has to be interpreted with caution as a GWAS with so few animals has very low power. Hence, the association reported here can only be taken as an indication on the presence of a genetic locus on chromosome 27 affecting PNST in Holstein dairy cattle and must be followed up by more powerful studies.

## Conclusions

Bovine PNSTs morphologically resemble schwannomatosis in humans. Danish Holstein cattle have a significantly higher prevalence of PNSTs than the other Danish dairy breeds as well as there were significant effect of sire and grand sire on the prevalence in the Holstein breed thus indicating a hereditary disposition. However, more animals need to be examined in order to determine the mode of inheritance and the involvement of specific loci or genes in the pathogenesis of bovine PNSTs.

## References

[1] Dukes TW, Bundza A, Corner AH (1982). Bovine neoplasms encountered in Canadian slaughterhouses: a summary. Can Vet J.

[2] Hamir AN (1985). An abattoir survey of neoplasms. Aust Vet J.

[3] Misdorp W (1967). Tumours in large domestic animals in the Netherlands. J Comp Pathol.

[4] Monlux AW, Anderson WA, Davis CL (1956). A survey of tumors occurring in cattle, sheep, and swine. Am J Vet Res.

[5] Wright BJ, Conner GH (1968). Adrenal neoplasms in slaughtered cattle. Cancer Res.

[6] Monlux AW, Davis CL (1953). Multiple schwannomas of cattle (nerve sheath tumors; multiple neurilemmomas; neurofibromatosis). Am J Vet Res.

[7] Nielsen AB, Jensen HE, Leifsson PS (2011). Immunohistochemistry for 2′,3’-cyclic nucleotide-3’-phosphohydrolase in 63 bovine peripheral nerve sheath tumors. Vet Pathol.

[8] Kleihues P, Cavenee WK (2000). Pathology and Genetics of Tumours of the Nervous System.

[9] Kwok K, Slimp JC, Born DE, Goodkin R, Kliot M, Berger MS, Prados MD (2005). Evaluation and management of benign peripheral nerve tumors and masses. Textbook of Neuro-Oncology.

[10] Plotkin SR, Blakeley JO, Evans DG, Hanemann CO, Hulsebos TJ, Hunter-Schaedle K, Kalpana GV, Korf B, Messiaen L, Papi L, Ratner N, Sherman LS, Smith MJ, Stemmer-Rachamimov AO, Vitte J, Giovannini M (2011). Update from the 2011 International Schwannomatosis Workshop: From genetics to diagnostic criteria. Am J Med Genet A.

[11] Carroll SL, Stonecypher MS (2005). Tumor suppressor mutations and growth factor signaling in the pathogenesis of NF1-associated peripheral nerve sheath tumors - II. The role of dysregulated growth factor signaling. J Neuropathol Exp Neur.

[12] Harder A, Wesemann M, Hagel C, Schittenhelm J, Fischer S, Tatagiba M, Nagel C, Jeibmann A, Bohring A, Mautner VF, Paulus W (2012). Hybrid neurofibroma/schwannoma is overrepresented among schwannomatosis and neurofibromatosis patients. Am J Surg Pathol.

[13] Sestini R, Bacci C, Provenzano A, Genuardi M, Papi L (2008). Evidence of a four-hit mechanism involving SMARCB1 and NF2 in schwannomatosis-associated schwannomas. Hum Mutat.

[14] Sartin EA, Doran SE, Riddell MG, Herrera GA, Tennyson GS, Dandrea G, Whitley RD, Collins FS (1994). Characterization of naturally-occurring cutaneous neurofibromatosis in Holstein cattle - A disorder resembling neurofibromatosis type-1 in humans. Am J Pathol.

[15] Doughty FR (1977). Incidence of neurofibroma in cattle in abattoirs in New South Wales. Aust Vet J.

[16] Murcia PR, Delhon G, Gonzalez MJ, Vilas M, Ramos-Vara JA, De Las HM, Nordhausen RW, Uzal FA (2008). Cluster of cases of malignant schwannoma in cattle. Vet Rec.

[17] Canfield PJ, Doughty FR (1980). A study of virus-like particles present in bovine nerve sheath tumours. Aust Vet J.

[18] Doughty FR (1972). Virus particles in a bovine neurofibroma. Aust Vet J.

[19] Miller SA, Dykes DD, Polesky HF (1988). A simple salting out procedure for extracting DNA from human nucleated cells. Nucleic Acids Res.

[20] Purcell S, Neale B, Todd-Brown K, Thomas L, Ferreira MA, Bender D, Maller J, Sklar P, de Bakker PI, Daly MJ, Sham PC (2007). PLINK: a tool set for whole-genome association and population-based linkage analyses. Am J Hum Genet.

[21] MacCollin M, Chiocca EA, Evans DG, Friedman JM, Horvitz R, Jaramillo D, Lev M, Mautner VF, Niimura M, Plotkin SR, Sang CN, Stemmer-Rachamimov A, Roach ES (2005). Diagnostic criteria for schwannomatosis. Neurology.

